# Cattle manure application for 12 and 17 years enhanced depth distribution of soil organic carbon and X-ray computed tomography-derived pore characteristics

**DOI:** 10.1038/s41598-023-50110-7

**Published:** 2023-12-27

**Authors:** Anuoluwa Ojonoka Sangotayo, Poulamee Chakraborty, Sutie Xu, Sandeep Kumar, Peter Kovacs

**Affiliations:** 1 Department of Agronomy, Horticulture, and Plant Science, South Dakota State University, Brookings, SD 57007 USA; 2 Department of Plant, Soil, and Microbial Sciences, Michigan State University, East Lansing, MI 48824 USA

**Keywords:** Plant sciences, Environmental sciences, Physics

## Abstract

Long-term fertilizer application in row crops may influence soil pore characteristics, thereby impacting soil aggregation and structure. Therefore, understanding the influences on soil pore characteristics is useful for adopting suitable conservation practices. However, the impact of cattle manure and inorganic fertilizer application at varied rates on soil pore characteristics in the soil profile at a microscale level remains limited. This study quantifies the impacts of manure and inorganic fertilizer amendments under a corn (*Zea mays* L.)-soybean (*Glycine ma*x L.)-spring wheat (*Triticum aestivum)* rotation system on soil pore characteristics using the X-ray computed tomography (XCT). Treatments included: low manure (LM; 4.4 and 3.3 Mg ha^−1^), medium manure (MM; 27.4 and 18.7 Mg ha^−1^), high manure (HM; 54.8 and 37.4 Mg ha^−1^), medium fertilizer (MF; 136 kg N ha^−1^, 49 kg P_2_O_5_ ha^−1^, and 91.5 kg K_2_O ha^−1^), high fertilizer (HF; 204 kg N ha^−1^, 73.5 kg P_2_O_5_ ha^−1^, and 137.3 kg K_2_O ha^−1^), and control (CK), respectively, at Brookings (initiated in 2008) and Beresford (2003) in South Dakota. Four intact soil cores were collected from each treatment at 0–10, 10–20, 20–30, and 30–40 cm depths. Results showed that the HM treatment increased the SOC by 8–68% compared to the CK and MF at 0–20 cm at the study sites. Both HM and MM treatments increased the macroporosity and mesoporosity in 0–20 cm soil depths at both study sites. Treatment did not always improve soil pore characteristics below 20 cm soil depth. Additionally, a positive correlation was observed between the XCT-derived macroporosity, total number of macropores, and SOC for all the treatments. Therefore, this study encourages the adoption of the XCT technique in quantifying soil pore characteristics and suggests that long-term medium manure application enhances soil structure as compared to an equivalent inorganic fertilizer application.

## Introduction

The changes in soil pore structure may influence the soil microbial activity and this may enhance or adversely impact soil organic carbon (SOC) content^1^ which in the long run impacts the overall soil structure. The distribution of soil pore space is responsible for crucial processes like water storage, nutrient cycling, gas exchange with the atmosphere, and solute transport^2–5^. Therefore, it is important to understand the effects of different soil amendments on the soil structure. Manure is used for soil amelioration and soil health improvement^6^. The addition of cattle manure to the soils can improve soil physical attributes such as soil aggregate stability and reduce soil compaction by increasing the SOC content and total nitrogen content^7–10^. In addition, with the presence of earthworms in manure, which are the major decomposers of organic matter, there is a release of binding agents that increase aggregate stability and improve soil structure^11^. It is a cost-effective way to dispose of and reprocess waste from livestock production. However, the over-application of manure is unacceptable from many points of view including greenhouse gas emissions, and can be detrimental to the environment^12^. For instance, Manure-derived Na^+^ has also been proposed to act as a dispersion agent which may lower soil aggregate stability^13^, thereby deteriorating the soil pore characteristics. Therefore, the effectiveness of manure application in increasing the SOC depends on the manure application rate^14^, duration of continuous manure application, and manure quality^9^.

Inorganic fertilizers and manure application affect the soil physical environment by increasing the above-ground and root biomass due to the immediate supply of nutrients in sufficient quantities to crops^15,16^. Over several decades, large-scale production and application of inorganic fertilizers have tremendously increased and surpassed the use of manure to achieve the goal of grain yield enhancement^8^. However, the use of inorganic fertilizers is generally associated with the reduction in soil aeration, reduction of SOC content, lower aggregate stability, decreased movement of water through the soils, decreased structural stability, and increased risk of nutrient losses^17^. The physical and chemical effects of mineral fertilizers on soil structure further depend on fertilizer type and their rates of application. For example, a large amount of ammonium in fertilizers can aid the dispersal of clay particles, thereby adversely affecting soil aggregation. On the other hand, phosphatic fertilizer stimulates the precipitation of aluminum oxide, iron oxide, and phosphate which can promote aggregate stability^18^. Mustafa et al. reported an increase in aggregate stability enhances SOC content^19^. Also, Liu et al. observed that a combination of inorganic fertilizer and manure increased SOC to a greater soil depth^5^. Therefore, it would be interesting to quantify the impacts of the long-term application of inorganic fertilizers alone on the soil pore characteristics to a greater soil depth.

X-ray computed tomography (XCT) is an innovative technique for visualizing soil pore structures^20^. Results can be achieved more accurately and faster when compared to using soil water retention data to quantify porosity. It is an efficient method to evaluate the effect of productive rejuvenation on aggregate stability and to visualize and quantify the 3-D soil pore network of different soil types^21,22^. For instance, XCT can be used to identify soil macroporosity and mesoporosity, which are more sensitive to management practices such as the application of manure and fertilizer. The use of XCT to identify the complex geometry of soil macropores in the order of a few micrometers allows correlating pore characteristics to water transport, as well as other physical properties of soils. The XCT scanning technique has been used to compare the pore structure of manure and inorganic fertilizer-treated soils. Zhou et al. used the micro-CT scanning technique to quantify soil porosity and found that soils treated with the combination of inorganic fertilizer and organic manure had greater intra- and inter-aggregate pores which significantly improved their saturated hydraulic conductivity, compared to that of the non-fertilized soils^17^. Despite the application of the XCT technique in studying soil pores under diverse management scenarios, studies involving the use of XCT to study soil pore systems at various soil depth after long-term manure and fertilizer applications at different rates in row crops are limited.

Studies have reported an improvement in soil physical attributes with manure application in combination with inorganic fertilizers as compared to unfertilized soils^23–25^. However, comparatively limited information is available on the long-term impact of only manure or only inorganic fertilizers at different rates on XCT-derived soil pore characteristics especially unique properties like tortuosity, fractal dimension, and average branch length. Two long-term (> 10 years) field experiments were utilized in this study to observe the changes in pore characteristics to 40 cm depth following the long-term application of manure and inorganic fertilizer at different rates. The present study is based on the hypothesis that long-term manure application may improve soil pore characteristics to a depth of 40 cm as compared to long-term inorganic fertilizer application. The specific objectives of the study are to (1) assess the impacts of different rates of only manure and only inorganic fertilizer on XCT-derived soil pore characteristics, soil organic carbon (SOC), and total nitrogen (TN) to a depth of 40 cm (2) examine the correlations between SOC and various XCT-derived soil pore-characteristics.

## Results

### Impact of manure and inorganic fertilizer application on Soil Organic Carbon (SOC) and Total Nitrogen content (TN)

**Figure 1:**
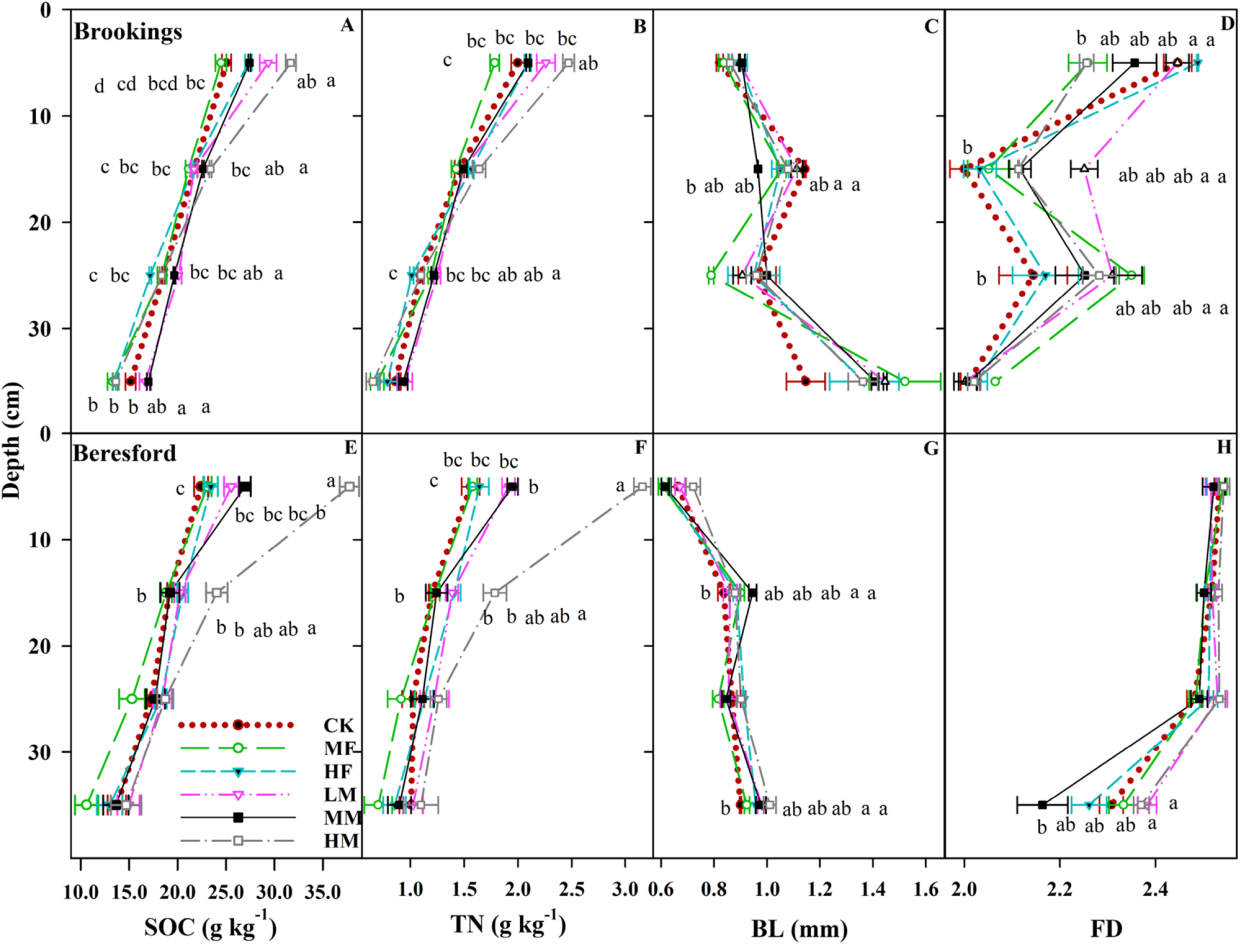
Soil depth and long-term nutrient application effects on soil organic carbon (SOC), total nitrogen (TN), branch length (BL), and fractal dimension (FD). Nutrient applications include medium fertilizer (MF), high fertilizer (HF), low manure (LM), medium manure (MM), high manure (HM) rate applications, and control (CK) at Brookings (**A**–**D**) and Beresford sites (**E**–**H**). Different lower-case letters indicate statistically significant different treatments at *p* ≤ 0.05 level at each depth.

The SOC and TN content at 0–10, 10–20, 20–30, and 30–40 cm depths for Brookings and Beresford sites are presented in Table 1 and Fig. 1. At Brookings site, significant treatment × depth interaction was observed for the SOC contents (Fig. 1A). The LM, MM, and HM treatments increased the SOC content as compared to the MF by 19.6, 11.8, and 29.4%, respectively, at 0–10 cm. At 10–20 cm, the HM increased SOC content by 7.8–10.9% as compared to CK, MF, HF, and LM. At 20–30 cm, the LM treatment increased SOC content by 8.6, 9.2, and 16.9% as compared to the CK, MF, and HM, respectively. At 30–40 cm, the LM and MM increased SOC contents by 22.1–27.8% when compared to the MF, HF, and HM. At Brookings site, the TN contents were influenced by the soil depth (Table 1). The TN content at 0–10 cm was 1.4, 1.9, and 2.6 times higher than TN contents at 10–20 cm, 20–30 cm, and 30–40 cm, respectively. In addition, the TN content at 10–20 cm was 1.3 times higher than the 20–30 cm depth and 1.4 times higher at 20–30 cm when compared to 30–40 cm, at Brookings site.

**Table 1:** Soil organic carbon (SOC), total nitrogen (TN), XCT-derived average branch length (BL), tortuosity (*τ*), degree of anisotropy (DA), and fractal dimension (FD) as influenced by long-term medium fertilizer (MF), high fertilizer (HF), low manure (LM), medium manure (MM), high manure (HM) rate applications, and control (CK) as a function of treatment and soil depths.

	SOC	TN	BL	Tortuosity (τ)	DA	FD
	(g kg^−1^)	(g kg^−1^)	(mm)			
Brookings site						
Treatment						
CK	20.1^a^	1.4^a^	1.019^a^	1.284^a^	0.306^a^	2.131^a^
MF	19.3^a^	1.3^a^	1.051^a^	1.285^a^	0.245^a^	2.121^a^
HF	19.9^a^	1.4^a^	1.066^a^	1.288^a^	0.261^a^	2.142^a^
LM	21.9^a^	1.5^a^	1.091^a^	1.273^a^	0.293^a^	2.158^a^
MM	21.7^a^	1.4^a^	1.070^a^	1.281^a^	0.320^a^	2.197^a^
HM	21.8^a^	1.5^a^	1.059^a^	1.281^a^	0.286^a^	2.166^a^
Depth (cm)						
0–10	27.5^a^	2.1^a^	0.870^c^	1.271^b^	0.294^a^	2.375^a^
10–20	22.0^b^	1.5^b^	1.068^b^	1.265^b^	0.323^a^	2.090^c^
20–30	18.7^c^	1.1^c^	0.928^c^	1.298^a^	0.229^b^	2.251^b^
30–40	14.8^d^	0.8^d^	1.371^a^	1.295^a^	0.294^a^	2.019^c^
Analysis of variance (P > *F)*						
Treatments	0.532	0.861	0.978	0.779	0.188	0.943
Depth	< 0.001	< 0.001	< 0.001	< 0.001	0.002	< 0.001
Trt × Depth	0.033	0.308	0.030	0.025	0.042	0.003
Beresford site						
Treatment						
CK	18.2^ab†^	1.2^b^	0.818^a^	1.209^a^	0.644^a^	2.460^a^
MF	16.9^b^	1.1^b^	0.812^a^	1.207^a^	0.654^a^	2.466^a^
HF	18.8^ab^	1.3^b^	0.844^a^	1.210^a^	0.583^a^	2.451^a^
LM	19.8^ab^	1.4^ab^	0.842^a^	1.216^a^	0.623^a^	2.487^a^
MM	19.4^ab^	1.3^b^	0.846^a^	1.211^a^	0.650^a^	2.420^a^
HM	23.8^a^	1.8^a^	0.878^a^	1.211^a^	0.631^a^	2.495^a^
Depth (cm)						
0–10	26.5^a^	2.0^a^	0.650^c^	1.228^a^	0.684^a^	2.529^a^
10–20	20.3^b^	1.4^b^	0.884^b^	1.221^a^	0.704^a^	2.515^a^
20–30	17.6^b^	1.1^bc^	0.867^b^	1.200^b^	0.697^a^	2.505^a^
30–40	13.4^c^	0.9^c^	0.958^a^	1.195^b^	0.439^b^	2.303^b^
Analysis of variance (P > *F)*						
Treatments	< 0.001	0.002	0.752	0.880	0.669	0.452
Depth	< 0.001	< 0.001	< 0.001	< 0.001	< 0.001	< 0.001
Trt × Depth	< 0.001	< 0.001	0.009	0.370	0.193	0.007

^†^Mean values within the same column followed by different small letters for each site are significantly different at *p* ≤ 0.05 for treatment.

At Beresford site, significant treatment × depth interactions were observed for both SOC and TN contents (Fig. 1E,F). The HM increased SOC and TN content by 40.1–93.8% as compared to other treatments, at 0–10 cm. At 10–20 cm, the HM increased SOC content by 25 and 27.7% as compared to the CK and MF, respectively. In addition, the HM treatment increased TN content as compared to the CK, MF, and MM treatments at 10–20 cm soil depth. There were no treatment impacts at 20–30 and 30–40 cm depth for SOC and TN contents at Beresford site.

### Impact of manure and inorganic fertilizer application on XCT-derived pore characteristics

The XCT-derived total number of pores (TP), the total number of macropores (Tmacro), and the total number of mesopores (Tmeso) for Brookings and Beresford sites are presented in Tables 2 and 3. Treatments by depth interactions were significant for the TP, Tmacro, and Tmeso in Brookings site (Tables 2 and 3). Where the HF treatment had 2.3 and 2 times lower TP than the MM and LM at 0–10 cm. At 10–20 cm, the MM and HM increased the TP 1.4–2.0 times more than the CK and HF. At 20–30 cm, the HM and MF increased the TP 1.4–2.0 times more than the CK, HF, and MM. However, treatment did not impact TP at 30–40 cm. For the Tmacro, the LM and MM increased Tmacro 1.7–2.0 times more than the MF and HF, at 0–10 cm. At 10–20 cm, the MM increased Tmacro was 1.5–1.9 times more than the CK, MF, and HF. In addition, the HM increased the Tmacro 2.7, 1.6, and 2.5 times more than the CK, LM, and MM, respectively, at 20–30 cm, at Brookings site. However, treatment did not impact Tmacro at 30–40 cm. For the Tmeso, the MM increased the Tmeso by 1.7, 1.8, 2.3, and 1.7 times more than the CK, MF, HF, and HM, respectively, at 0–10 cm at Brookings site. At 10–20 cm, the MM increased the Tmeso by 1.5–2.0 times more than the CK, MF, HF, LM, and HM. At 20–30 cm, the MF and HF increased the Tmeso by 1.5–2.0 times more than the CK, LM, and MM at Brookings site. However, treatment did not impact Tmeso at 30–40 cm*,* in Brookings site.

**Table 2:** XCT-derived macroporosity, mesoporosity, porosity total number of pores, macropores, and mesopore as influenced by long-term medium fertilizer (MF), high fertilizer (HF), low manure (LM), medium manure (MM), high manure (HM) rate applications, and control (CK) as a function treatment and soil depth.

	Total no. of pores	Total no. of macropores	Total no. of mesopores	Porosity (cm^3^cm^−3^)	Macroporosity (cm^3^cm^−3^)	Mesoporosity (cm^3^cm^−3^)
Brookings site						
Treatment						
CK	858^a﻿†^	113^a^	745^a^	0.010^a^	0.007^ab^	0.003^a^
MF	977^a^	116^a^	861^a^	0.009^a^	0.005^b^	0.002^a^
HF	839^a^	113^a^	726^a^	0.012^a^	0.008^ab^	0.002^a^
LM	1256^a^	166^a^	1089^a^	0.013^a^	0.010^ab^	0.003^a^
MM	1421^a^	168^a^	1253^a^	0.017^a^	0.010^a^	0.004^a^
HM	1111^a^	152^a^	978^a^	0.014^a^	0.009^ab^	0.003^a^
Depth (cm)						
0–10	2411^a^	299^a^	2111^a^	0.020^a^	0.013^a^	0.007^a^
10–20	941^b^	125^b^	815^b^	0.010^b^	0.010^b^	0.002^bc^
20–30	528^c^	61^c^	466^c^	0.015^b^	0.006^c^	0.002^b^
30–40	428^c^	66^c^	375^c^	0.005^c^	0.003^d^	0.001^c^
Analysis of variance (P > *F)*						
Treatments	0.4170	0.4398	0.4017	0.1489	0.0358	0.4757
Depth	< 0.0001	< 0.0001	< 0.0001	< 0.0001	< 0.0001	< 0.0001
Trt × Depth	< 0.0001	< 0.0001	< 0.0001	< 0.0001	< 0.0001	< 0.0001
Beresford site						
Treatment						
CK	2176^b†^	190^a^	1986^b^	0.020^c^	0.014^b^	0.006^c^
MF	2609^ab^	188^a^	2421^ab^	0.024^bc^	0.016^ab^	0.008^bc^
HF	2911^a^	235^a^	2676^a^	0.024^bc^	0.014^b^	0.010^ab^
LM	2703^ab^	214^a^	2488^ab^	0.028^ab^	0.019^a^	0.009^abc^
MM	2888^a^	237^a^	2652^a^	0.025^bc^	0.017^ab^	0.008^abc^
HM	2965^a^	242^a^	2723^a^	0.031^a^	0.020^a^	0.011^a^
Depth (cm)						
0–10	3128^a^	258^a^	2869^a^	0.029^a^	0.020^a^	0.010^a^
10–20	2836^a^	230^ab^	2606^a^	0.025^ab^	0.018^ab^	0.008^a^
20–30	2725^a^	199^bc^	2525^a^	0.026^ab^	0.015^bc^	0.008^a^
30–40	2145^b^	182^c^	1963^b^	0.023^b^	0.014^c^	0.008^a^
Analysis of Variance (P > *F)*						
Treatments	0.0036	0.0318	0.0051	< 0.0001	< 0.0001	< 0.0001
Depth	< 0.0001	< 0.0001	< 0.0001	0.0035	< 0.0001	0.2291
Trt × Depth	0.9915	0.9610	0.9810	< 0.0001	0.0062	0.0037

^†^Mean values within the same column followed by different small letters for each site are significantly different at *p* ≤ 0.05 for treatment.

**Table 3:** XCT-derived total number of pores, macropores, and mesopores as influenced by long-term medium fertilizer (MF), high fertilizer (HF), low manure (LM), medium manure (MM), high manure (HM) rate applications, and control (CK) across 0–40 cm depth.

	Total no. of pores				Total no. of macropores				Total no. of mesopores			
	0–10	10–20	20–30	30–40	0–10	10–20	20–30	30–40	0–10	10–20	20–30	30–40
	Depth (cm)				Depth (cm)				Depth (cm)			
Brookings site												
Treatment												
CK	2075^bc﻿†^	672^c^	345^c^	341^a^	279^ab^	89^c^	35^d^	49^a^	1796^bc^	583^c^	310^c^	292^a^
MF	2005^bc^	826^bc^	691^a^	387^a^	234^b^	89^c^	76^ab^	64^a^	1778^bc^	737^bc^	615^a^	323^a^
HF	1555^c^	778^c^	626^ab^	401^a^	205^b^	115^bc^	66^abc^	65^a^	1349^c^	661^c^	559^a^	337^a^
LM	3144^ab^	928^bc^	454^bc^	498^a^	389^a^	138^ab^	59^bcd^	81^a^	2755^ab^	790^bc^	396^bc^	417^a^
MM	3528^a^	1358^a^	405^c^	395^a^	407^a^	170^a^	38^ cd^	59^a^	3121^a^	1187^a^	367^c^	337^a^
HM	2159^bc^	1088^ab^	646^a^	551^a^	284^ab^	152^ab^	94^a^	79^a^	1875^bc^	936^b^	552^ab^	547^a^
Analysis of variance (*p* > F)												
	0.0009	< 0.0001	< 0.0001	0.1279	0.0010	< 0.0001	< 0.0001	0.2465	0.0009	< 0.0001	< 0.0001	0.0808
Beresford site												
Treatment												
CK	2732^a†^	2372^a^	2303^a^	1297^b^	233^a^	195^a^	168^a^	164^a^	2499^a^	2177^a^	2135^a^	1133^b^
MF	2997^a^	2859^a^	2489^a^	2091^ab^	245^a^	189^a^	167^a^	153^a^	2752^a^	2670^a^	2323^a^	1938^ab^
HF	3327^a^	3096^a^	2979^a^	2240^ab^	253^a^	262^a^	232^a^	192^a^	3074^a^	2834^a^	2748^a^	2048^ab^
LM	3133^a^	2782^a^	2669^a^	2226^ab^	244^a^	219^a^	210^a^	182^a^	2889^a^	2562^a^	2450^a^	2043^ab^
MM	3125^a^	2938^a^	2925^a^	2565^a^	303^a^	273^a^	191^a^	178^a^	2822^a^	2665^a^	2374^a^	2387^a^
HM	3453^a^	2970^a^	2983^a^	2452^a^	272^a^	243^a^	229^a^	222^a^	3810^a^	2727^a^	2754^a^	2230^a^
Analysis of variance (*p* > F)												
	0.5445	0.1575	0.5311	0.0251	0.5733	0.4459	0.336	0.1420	0.5699	0.2304	0.5577	0.0268

^†^Mean values within the same column followed by different small letters for each site are significantly different at *p* ≤ 0.05.

Considering treatment as the main effect, the MM, HM, and HF treatment increased the TP compared to the CK treatment at Beresford site. Considering depth as the main effect, the TP and Tmacro values were higher at 0–10 cm compared to the 30–40 cm in Beresford site (Table 2). The TP at 30–40 cm was 1.5, 1.32, and 1.27 times lower than at 0–10 cm, 10–20 cm, and 20–30 cm, respectively. The Tmacro at 0–10 cm was 1.3 and 1.4 times higher than the Tmacro at 20–30 cm and 30–40 cm, respectively. The Tmacro at 10–20 cm was 1.3 times higher than the Tmacro at 30–40 cm.

**Figure 2:**
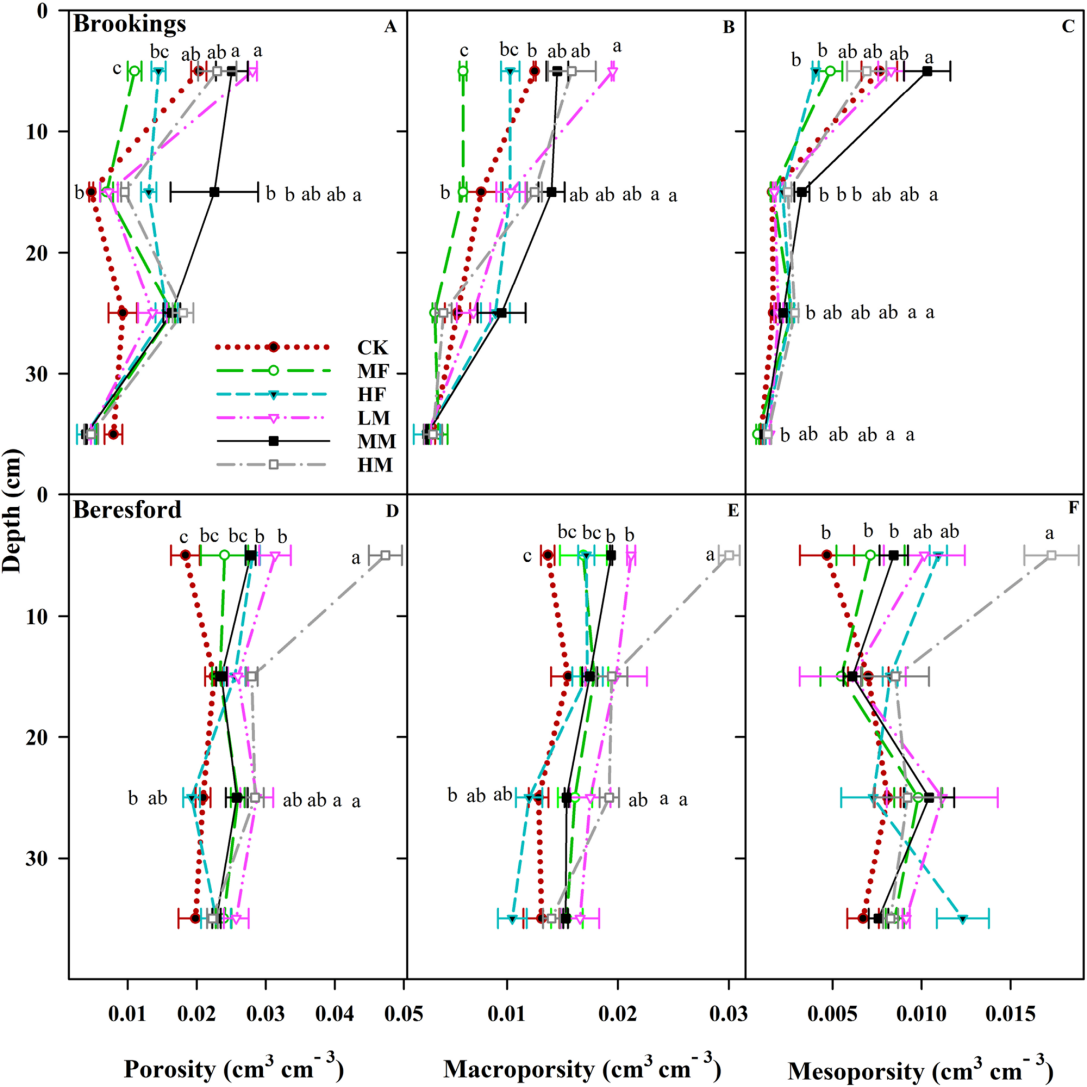
Soil depth and long-term nutrient application effects on porosity (cm cm^−3^), macroporosity (cm cm^−3^), and mesoporosity (cm cm^−3^). Nutrient applications include medium fertilizer (MF), high fertilizer (HF), low manure (LM), medium manure (MM), high manure (HM) rate applications, and control (CK) at Brookings (**A**–**C**) and Beresford sites (**D**–**F**). Different lower-case letters indicate statistically significant different treatments at *p* = 0.05 level at each depth.

The XCT-derived porosity, macroporosity, and mesoporosity in both sites are presented in Fig. 2. Treatments by depth interactions were mainly significant for porosity, macroporosity, and mesoporosity in both sites (Fig. 2). At Brookings site, the porosity for the LM, MM, and HM treatments was 1.6 to 2.6 times higher than the MF and HF at 0–10 cm (Fig. 2A). At 10–20 cm, the MM treatment increased soil porosity by 3.1–4.4 times more than the CK, MF, and LM. Treatment × depth interaction was not significant for porosity at lower depths (20–40 cm). At Brookings site, the macroporosity values of the LM treatment were 1.7, 3.3, and 2.0 times higher than the CK, MF, and HF treatments, respectively, at 0–10 cm (Fig. 2B). In addition, the MM and HM treatments showed 2.3 and 2.0 times higher macroporosity than the MF treatment, respectively, at 10–20 cm. Treatment × depth interaction was not significant for macroporosity at lower depths (20–40 cm). The MM for mesoporosity at Brookings site was 2.0 and 2.5 times higher than MF and HF, respectively, at 0–10 cm (Fig. 2C). At 10–20 cm, the MM treatment increased the soil mesoporosity compared to the CK, MF, and LM. At 20–30 cm, the MF and HM increased the soil mesoporosity by 50% compared to the CK. In addition, at 30–40 cm, the LM and HM treatment increased the soil mesoporosity as compared to the MF treatments at Brookings site.

At Beresford site, the HM increased soil porosity as compared to the CK, MF, HF, LM, and MM at 0–10 cm; no differences were observed at 10–20 cm (Fig. 2D). At 20–30 cm, the LM and HM treatments increased the soil porosity by 53 and 47% as compared to the HF; no differences were observed at 30–40 cm. At Beresford site, the HM treatment showed 1.4–2.1 times higher macroporosity than other treatments at 0–10 cm (Fig. 2E). At 20–30 cm, the HM increased macroporosity 1.6 times more than the HF. Treatment × depth interaction was not significant for macroporosity at 10–20 cm and 30–40 cm. The HM for mesoporosity was 2.1, 2.4, and 3.4 times higher than the MM, MF, and CK, respectively, at 0–10 cm (Fig. 2F). Treatment × depth interaction was not significant for mesoporosity at lower depths (10–40 cm).

The XCT-derived average branch length (BL), tortuosity (τ), degree of anisotropy (DA), and fractal dimension (FD) in both sites are presented in Table 4 and Fig. 1C,D,G,H. Treatments by depth interactions were mainly significant for the average branch length (BL), tortuosity (τ), degree of anisotropy (DA), and fractal dimension (FD) for Brookings site (Table 4 and Fig. 1C,D) and were significant for the BL and FD values in Beresford site (Fig. 1G,H). At Brookings site, the LM treatment for BL was 14.4% higher than the MM, at 10–20 cm soil depth. However, there were no significant differences observed at other depths. The MF increased tortuosity (τ) more than CK and LM by 4.8 and 5.7%, respectively at 10–20 cm. However, treatment did not impact other depths. In addition, the LM, MM, and CK increased the DA by 8.4–10.2% as compared to the MF and HF at 0–10 cm. At 10–20 cm, the MM increased the DA by 9.8–13.6% as compared to the CK, MF, and LM. However, treatment did not impact 20–40 cm depths. Also, the FD for the MM was 2 times higher than the MF at 20–30 cm soil depth. However, treatment did not impact other depths (Table 4). At Beresford site, the MM increased the BL by 13.1% as compared to the CK at 10–20 cm. At 30–40 cm, the HM increased BL by 9.8 and 12.2% as compared to the MF and CK, respectively. However, treatment did not impact the BL at 0–10 and 20–30 cm (Fig. 1G). The LM and HM treatments for FD were 10.2% and 9.7% higher than the MM at Beresford site for 30–40 cm depth. However, treatment did not impact the FD at 0–30 cm (Fig. 1H).

**Table 4:** XCT-derived tortuosity **(***τ*), degree of anisotropy (DA) as influenced by long-term medium fertilizer (MF), high fertilizer (HF), low manure (LM), medium manure (MM), high manure (HM) rate applications, and control across 0–40 cm depth.

	Tortuosity (τ)				DA			
	0–10	10–20	20–30	30–40	0–10	10–20	20–30	30–40
	Depth(cm)				Depth(cm)			
Brookings site								
Treatment								
CK	1.28^a﻿†^	1.24^b^	1.29^a^	1.32^a^	0.68^ab^	0.75^a^	0.72^a^	0.44^ab^
MF	1.25^a^	1.30^a^	1.31^a^	1.29^a^	0.70^a^	0.68^a^	0.70^a^	0.41^ab^
HF	1.27^a^	1.28^ab^	1.30^a^	1.31^a^	0.60^b^	0.66^a^	0.70^a^	0.37^b^
LM	1.28^a^	1.23^b^	1.31^a^	1.28^a^	0.72^a^	0.72^a^	0.69^a^	0.48^ab^
MM	1.27^a^	1.27^ab^	1.29^a^	1.29^a^	0.70^a^	0.71^a^	0.66^a^	0.52^a^
HM	1.28^a^	1.27^ab^	1.30^a^	1.28^a^	0.70^ab^	0.70^a^	0.72^a^	0.40^b^
Analysis of variance (*p* > F)								
	0.061	0.016	0.333	0.549	0.001	0.578	0.817	0.009
Beresford site								
Treatment								
CK	1.22^a﻿†^	1.22^a^	1.19^a^	1.19^b^	0.30^a^	0.35^a^	0.25^ab^	0.32^a^
MF	1.22^a^	1.22^a^	1.19^a^	1.19^b^	0.27^a^	0.23^a^	0.16^b^	0.33^a^
HF	1.23^a^	1.22^a^	1.18^a^	1.20^a^	0.23^a^	0.33^a^	0.18^ab^	0.30^a^
LM	1.22^a^	1.23^a^	1.20^a^	1.20^a^	0.36^a^	0.37^a^	0.24^ab^	0.21^a^
MM	1.23^a^	1.21^a^	1.21^a^	1.20^ab^	0.37^a^	0.29^a^	0.32^a^	0.30^a^
HM	1.23^a^	1.22^a^	1.19^a^	1.21^a^	0.24^a^	0.37^a^	0.22^ab^	0.31^a^
Analysis of variance (*p* > F)								
	0.966	0.212	0.533	0.001	0.078	0.253	0.037	0.333

^†^Mean values within the same column followed by different small letters for each site are significantly different at *p* ≤ 0.05.

Considering depth as the main effect, the τ, and DA showed varying impacts across the soil depths in the Beresford site (Table 1). The τ and DA showed significant depth effect and the τ values for 0–10 cm and 10–20 cm was higher than the τ values for 20–30 cm and 30–40 cm by 1.8 to 2.8%, respectively. Also, the DA at 30–40 cm was lower than 0–10, 10–20, and 20–30 cm, by 8.8 to 60.4% at Beresford site.

### Correlations between SOC, TN content, and various XCT-derived soil pore characteristics.

**Figure 3:**
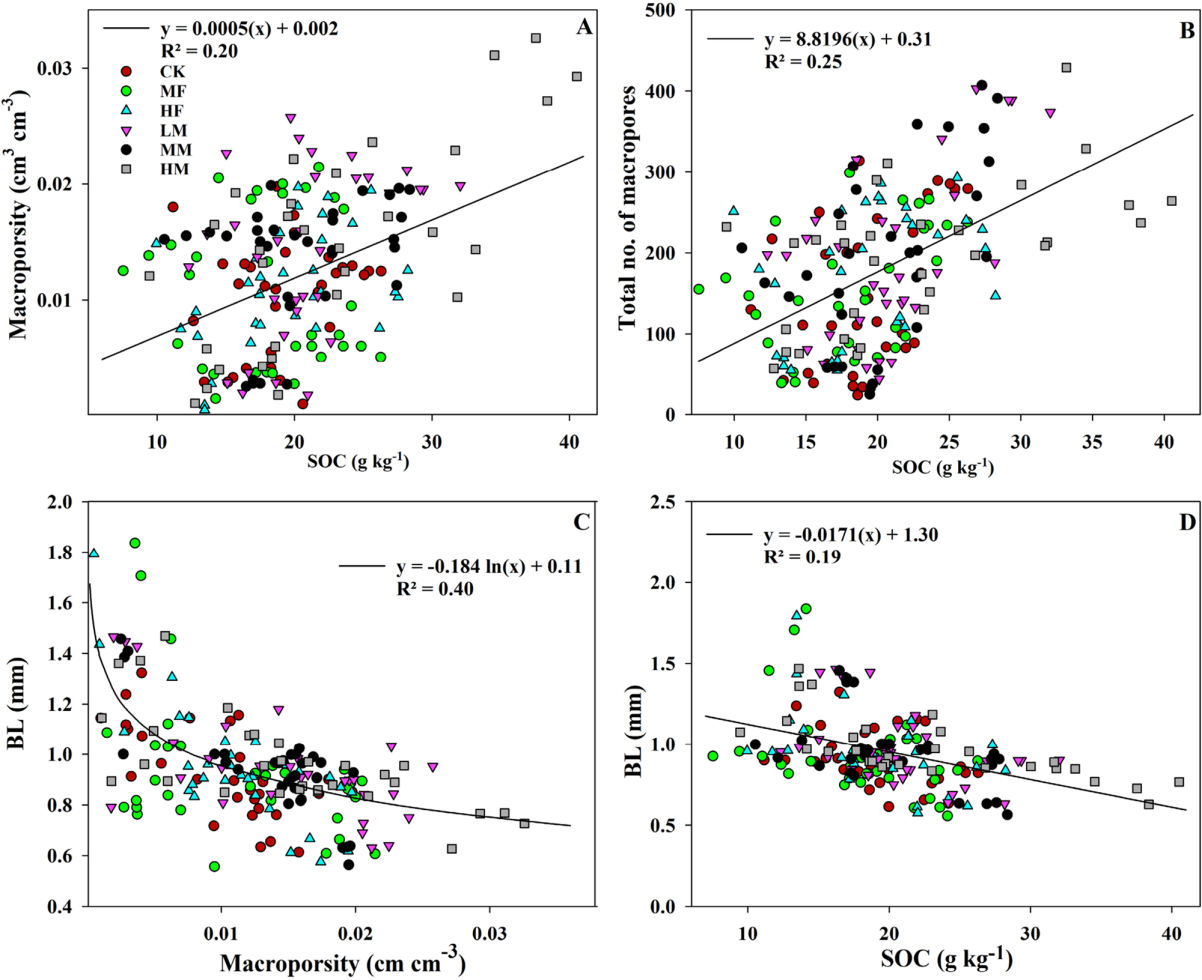
Relationship between (**A**) SOC content and macroporosity, (**B**) SOC content and the total number of macropores, (**C**) branch length (BL, *mm*) and macroporosity, and (**D**) SOC and BL. Nutrient applications include medium fertilizer (MF), high fertilizer (HF), low manure (LM), medium manure (MM), high manure (HM) rate applications, and control (CK) at Brookings and Beresford sites.

**Figure 4:**
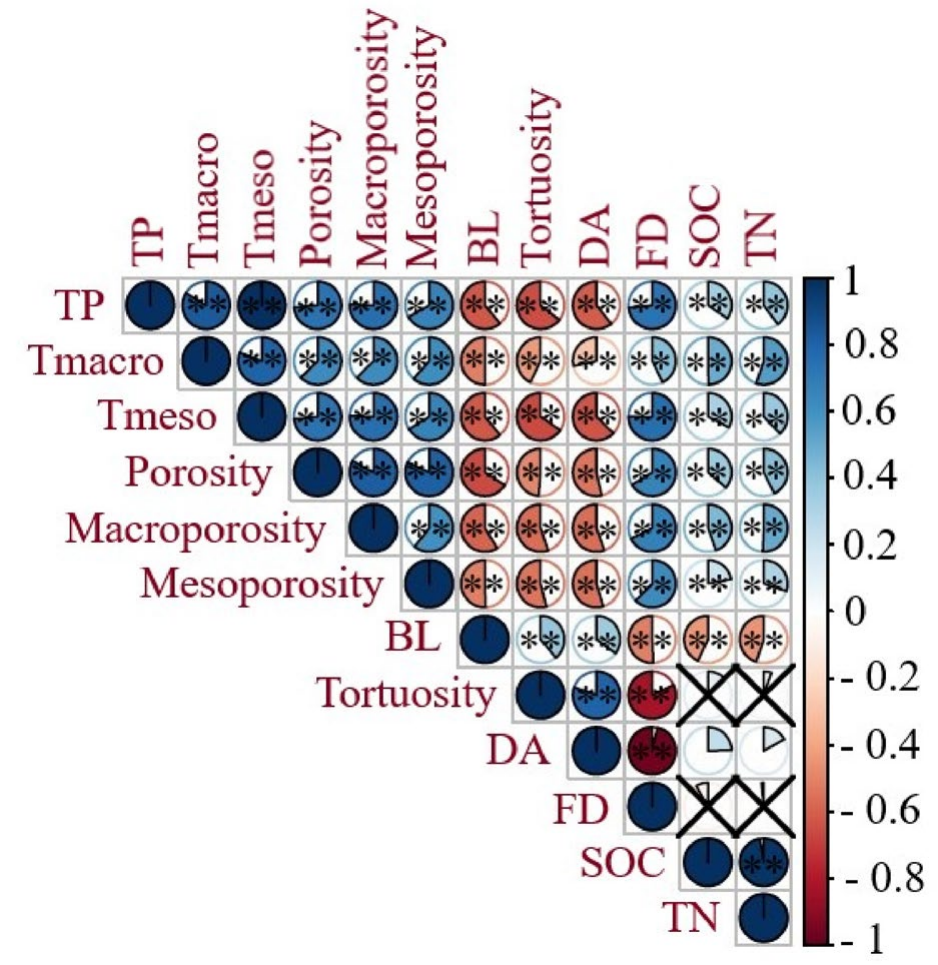
Correlation between SOC, TN content, and XCT-derived soil pore characteristics as influenced by long-term medium fertilizer (MF), high fertilizer (HF), low manure (LM), medium manure (MM), high manure (HM) rate applications, and control (CK) across 0–40 cm soil depth, at Brookings and Beresford sites. **Significant at the < 0.01 level.

**Figure 5:**
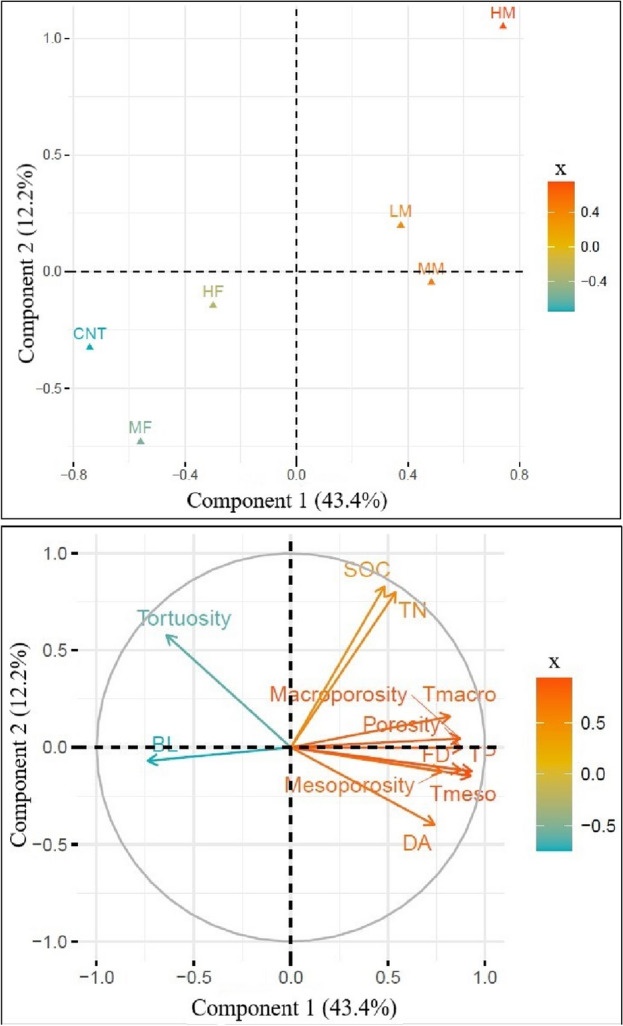
Principal component analysis (PCA) of the soil organic carbon (SOC), total nitrogen TN content, and XCT-derived soil pore characteristics (macroporosity, mesoporosity, porosity, fractal dimension—FD, degree of anisotropy—DA, total number of pores—TP, total number of macropores—Tmacro, total number of mesopores—Tmeso) as influenced by long-term medium fertilizer (MF), high fertilizer (HF), low manure (LM), medium manure (MM), high manure (HM) rate applications, and control (CK) across 0–40 cm soil depth, at Brookings and Beresford sites.

The SOC content is positively correlated with the TN content, macroporosity, and total number of macropores (Tmacro) and is negatively correlated to the average branch length (BL) (Figs. 3 and 4). Therefore, the differences observed in SOC content amongst LM, MM, HM, MF, HF, and CK treatments might indicate SOC content can directly impact the XCT-derived soil pore characteristics. Principal component analysis (PCA) was performed to evaluate how the measured XCT-derived parameters and SOC and TN values were distributed among the six treatments (Fig. 5). The PC1 and PC2 explained 12% and 43% of the variation, accounting for 55% of the total variance. Different groups formed across the two principal component axes indicated the effects of different manure and fertilizer application rates on soil pore characteristics. Specifically, the TP, Tmacro, Tmeso, porosity, macroporosity, mesoporosity, and FD were more relevant to the PC1, whereas SOC, TN, and tortuosity values were more important to the PC2. The PC1 differentiated the control and fertilizer treatments from the manure treatments. The PC2 better differentiated the three manure treatments (LM, MM, and HM) probably because of higher SOC and TN in HM. The contribution of XCT-measured soil parameters to PC1 emphasized the positive influence of manure treatments on soil pore characteristics.

## Discussion

In this study, high manure application rate was found to have the largest impact on the SOC and TN contents as compared to the medium inorganic fertilizer application rate at the surface depth (0–10 cm) of both sites. It is likely due to the direct addition of larger amounts of organic matter and total nitrogen in the form of animal manure, and indirectly through increased stubbles and root residues^26,27^. Ozlu et al. observed that a higher manure application rate increases the soil organic matter and total nitrogen when compared to a lower manure application rate^7^. Therefore, an increasing amount of manure would increase SOC and TN content, but a moderate application of manure would be more beneficial for the long term over a high manure application rate^28^. On the other hand, inorganic fertilizers indirectly influence SOC contents by increasing crop yields, thereby impacting biomass inputs, and increasing the return of crop residues to the soil^29^. This might be the reason for the observed high values of SOC and TN for HF plots at both sites in our study. However, the long-term use of inorganic fertilizer and high manure application rate may increase the risk of environmental pollution thereby negatively impacting the environment^30,31^. The highest SOC content was measured at the surface depth. This may be because of supplied nutrients at the surface depth and decreasing root biomass with depth increase^5,32^. We also observed that the different manure applications improved the SOC to a greater soil depth at Brooking site. This may be due to earthworms burrowing through the soil causing the SOC content to move to a greater soil depth^33^.

In addition, the application of manure was found to improve the soil pore characteristics compared to the application of inorganic fertilizer. Similar results were observed by Zhou et al. and Fang et al.^17,34^. Manure can enhance soil microbial activity and increase microbial metabolites^35^. Soil microorganisms bind aggregates by various mechanisms, such as adsorption, physical morass, and cementation by excreted mucilaginous products^36^. Prominent among the many microbial products are polysaccharides. The release of polysaccharides and bacterial gum will help bind soil particles and improve soil aggregation^37^. Therefore, promoting the formation of soil aggregate increases the total number of macropores and mesopores. Also, the presence of earthworms in manure helps decompose organic matter, and release binding agents which increase aggregate stability and improve soil structure^11^. Macropores play an important role in crucial soil physical processes, like, water infiltration and solute transport^38^. In addition, macropores provide a faster route for root growth^34^. Manure application helped improve the number of macropores in our research, which is in agreement with the finding of Singh et al. ^20^. A positive correlation between the number of macropores and macroporosity in this study indicates that the formation of numerous macropores per unit volume of soil under manure treatment, mostly because of the increase in soil aggregation, enhanced the macroporosity. Zhang et al. also observed a strong correlation between the total number of macropores and macroporosity^39^ in this study. When observing the depth impact, a decrease in the number of pores with an increase in soil depth was observed in this study which was also reported by^40^. This could be because the impacts of the application of manure and inorganic fertilizer decrease with increasing depth.

The FD ranged from 2.0 to 2.5, where the FD value at 30–40 cm soil depth was lower than the other depths (0–30 cm). In general, the various rates of inorganic fertilizer and manure application had little to no impact on soil FD at different depths. Fang et al. 2021, concluded similar effects as well^41^. Rivier et al. reported an increased FD with manure application which was similar to our findings in the Brookings site, where the application of MM increased FD as compared to MF treatments at 20–30 cm soil depth^42^. Higher FD values can explain a more complex pore structure^43^ which can contribute to soil fertility^44^. Therefore, our study shows that MM application improved the formation and stabilization of soil structure.

According to Arora et al. 2012, higher soil τ values indicate lower soil pore connection and vice versa^45^. In addition, Pires et al. suggested that a higher value of τ with an increased number of pores at lower depth favors the formation of pores of irregular shape^46^. Thereby influencing pore connectivity. In this study, there was no overall difference in soil τ among the different treatments regardless of the little impacts found at a greater soil depth. This can be because when τ was lower in inorganic fertilizer than manure application, the total number of pores was higher, resulting in increased interconnected pores.

Manure may promote aggregate stability by reducing soil wettability and swelling. This may be attributed to the unique properties of manure. Which is that it is inherently hydrophobic, or becomes so with dehydration, so that the organo-clay complex may have a reduced affinity for water^47^. The rearrangement of soil aggregates due to shrinkage and swelling can affect the soil pore characteristics, which can further impact the soil structure ^48^. To better understand the impacts of the various soil pore characteristics on the soil structure, observing the combined effects is necessary. For instance, as previously discussed, a decrease in tortuosity values indicates increased pore interconnectivity which may indicate soil degradation. Also, Hu et al. 2015, reported that high porosity and tortuosity may be related to a well-developed root system^49^. We observed higher soil tortuosity and porosity at 0–10 cm soil depth in Brookings. Therefore, indicating an improved root system.

Similar to previous studies, we observed a positive correlations between SOC content and the total no. of macropores and macroporosity^44,50^, showing that an increase in macroporosity might be the result of the increase in SOC under manure treatments.. Organic matter enhances soil aeration promoting soil biological activities which could positively enhance the total no. of macropores^39^ as macropores are formed by soil biological activity like earthworm burrowing and root growth^51^. Pagenkemper et al. reported that soil tortuosity (τ) explains the complexity of the soil pores, which was found to be negatively correlated to macroporosity, mesoporosity, and the total number of mesopores^52^. This could be explained by Vogel, 1997, who reported that as pore sizes increases pore connectivity decreases^4^.

## Materials and methods

### Study site

Sampling was conducted from two existing long-term studies located at South Dakota State University’s research farms. The first site was located at Felt Research Farm (44° 22′ 07.15″ N and 96° 47′ 26.45″ W) near Brookings, South Dakota on a well-drained Vienna soil (*Fine-loamy, mixed, frigid Udic Haploborolls*), and the second site was at Southeast Research Farm (43° 02′ 33.46″ *N* and 96° 53′ 55.78″ *W*) in Clay County, near Beresford, South Dakota, USA, on Egan soil (*Fine-silty, mixed, mesic Udic Haplustolls*). The long-term research was initiated in 2008 at Brookings and in 2003 at Beresford to study the effect of organic and inorganic fertilizer application rates on crop production and soil properties. The plot dimensions for Brookings site were 6 m by 18 m. The plots were nearly flat with a slope of < 1% and an elevation of 518 m. The experimental areas are in a humid continental climate having relatively humid summers and cold, snowy winters with a mean air temperature of 27.8 °C in the summer and − 15.8 °C in the winter, respectively. The mean annual precipitation was about 638 mm. The plots at Beresford site were established in nearly flat areas with a slope of < 1%, and an elevation of 390 m. The plot dimensions at Beresford were 5 m by 20 m. This experimental site was observed with a humid continental climate having relatively humid summers and snowy winters with a mean air temperature of 29.5 °C in the summer and − 13.6 °C in the winter, respectively. The mean annual precipitation was about 678 mm.

### Study treatments

Each study site included six treatments: The three manure application rates included were; low manure (LM) contained a quantity of manure rate based on the recommended phosphorous requirement, medium manure (MM) contained a quantity of manure rate based on recommended nitrogen requirement, and high manure (HM) contained a quantity of manure-based on double the recommended nitrogen requirement. The two inorganic fertilizer application rates included were; medium fertilizer (MF) contained the recommended fertilizer rate, high fertilizer (HF) contained a high fertilizer rate, and a control treatment (CK) which did not receive manure and fertilizer. Treatments were arranged in a randomized complete block design with four replicates. The cropping system was corn (*Zea mays* L.)-soybean [*Glycine max* (L.) Merr] rotation at both sites, until 2019. In 2020, spring wheat (*Triticum aestivum* L*.)* and cover crops (radish) were added to the cropping system, and it became a corn-soybean-spring wheat rotation system.

The manure and fertilizer application rates followed the South Dakota Fertilizer Recommendation Guide for the 3.7 Mg ha^−1^ spring wheat yield goal in 2020. Additional treatment details used in this study since 2003 can be found in ^53,54^. Beef dry and dairy solid manure were used at the Beresford and Brookings sites, respectively, to apply the target nutrient rates according to the treatments. The manure used was analyzed by certified commercial laboratories for nutrient concentration to determine application rates. No manure or fertilizer was applied before the soybean. In 2020, 4.4, 27.4, and 54.8 Mg ha^−1^ manure was applied at the Brookings site, while 3.3, 18.7, and 37.4 Mg ha^−1^ manure was applied at Beresford for the LM, MM, and HM treatments, respectively. Urea, mono ammonium phosphate, and potash fertilizer were used for the inorganic fertilizer. The inorganic fertilizer rate treatments included: 136 kg N ha^−1^, 49 kg P_2_O_5_ ha^−1^, and 91.5 kg K_2_O ha^−1^, respectively, for MF, and 204 kg N ha^−1^, 73.5 kg P_2_O_5_ ha^−1^, and 137.3 kg K_2_O ha^−1^, respectively, for HF. The study sites were tilled to a soil depth of 20 cm about 1–3 days before planting, to incorporate all the treatments added and residue left on the field.

### Soil sampling and sample preparation

A total of 192 (96 from each site) intact cores from six treatments, four soil depths (0–10, 10–20, 20–30, and 30–40 cm), and four replications from each site were collected in July 2020. Plexiglass cores (76.2 mm long and 76.2 mm in diameter, with a 3.2-*mm*-thick wall) were used for the sampling. These cores were extracted from the soil manually using a core sampler vertically inserted in the soil (Fig. 5). For each depth, the plexiglass cores were inserted leaving 11.9 mm of soil at the top and bottom to minimize disturbance to the intact soil sample. Soil cores were then trimmed using a serrated knife, sealed with plastic caps at both ends, labeled, and stored in plastic bags at 4 °C pending analysis. In the laboratory, soil cores were slowly saturated from the bottom and then drained at − 4.0 kPa using a low-tension table to remove water from macropores to improve image contrast for XCT scanning. Samples were secured at both ends with wooden caps and masking tape and stored in a cold room in preparation for scanning. The cores were transported in a cooler to the University of Missouri Veterinary Health Center in Columbia, MO for XCT scanning.

### X-ray computed tomography scanning and image analysis

Intact core samples were scanned using a Toshiba Aquilion 64 X-ray CT scanner to acquire images. About ten cores were placed horizontally on the scanner bench per time. To perform a spiral scanning with a peak voltage current of 135 kV and an X-ray tube current of 200 mA. The slice thickness was 0.28 mm, producing a resultant voxel size of (0.26 × 0.26 × 0.28) mm^3^. The entire sample was imaged with a field of view of 512 by 512 pixels. The captured XCT scanned data were exported as a stack of TIFF images and 3D visualization, image cropping, and segmentation were performed using the public domain processing software FIJI (*ImageJ software*) ^55–57^. Image slices that were subject to interference from the beam hardening were removed from the stack ^58^. Stacks were then cropped to obtain a region of interest (ROI) (60 mm in diameter and 60 mm in height). Stacks were pre-processed with a median three-dimensional filter (radius = 2.0 voxels) to reduce the possibility of reading noise, and contrast enhancement with saturated pixels of 0.4% was used to improve the contrast between the soil matrix and pores in the image. The images were converted to eight-bit and the segmentation process was carried out by the auto-local threshold algorithm of Phansalkar taking the parameter radius as 10, parameter 1(k) as 0.3, and parameter 2 (r) as 0.2 in Image*J* ^59^. The Phalsankar algorithm is a modification of Sauvola’s thresholding method to deal with low-contrast images^60^. This procedure resulted in a binary image in which pores and soil matrix were represented by white and black pixels, respectively. The binary image obtained from segmentation was then visually inspected to check the image quality and the presence of artifacts, and features made up of one voxel were removed to avoid classification of noise in further analysis^58^. Several definitions of macropores are available in the literature. For example, pores with an equivalent cylindrical diameter (ECD) larger than 1 mm^61^, larger than 1.2 mm^38^, and larger than 3 mm^62^ were considered as macropores. In this study, pores with ECD ≥ 1000 μm were considered as macropores. Macroporosity (> 1000 μm ECD) and coarse mesoporosity (150–1000 μm ECD) and total porosity, which is macroporosity plus coarse mesoporosity, were obtained as the ratio of the total volume of macropores and coarse mesopores, and all pores, respectively, to the volume of ROI.

#### Quantification of pore characteristics

To measure the pore characteristics, the “Particle Analyser” and “Skeletonize-3D” plugins available with BoneJ in *ImageJ* ^63^ were used. ImageJ was used to estimate the total number of pores (TP), total number of macropores (MP), and total number of mesopores. The skeletonized binary stack provided a summary of the tortuosity (τ), average branch length (BL, *mm*), fractal dimension (FD), and degree of anisotropy (DA) for each of the 192 intact soil cores.

**Figure 6:**
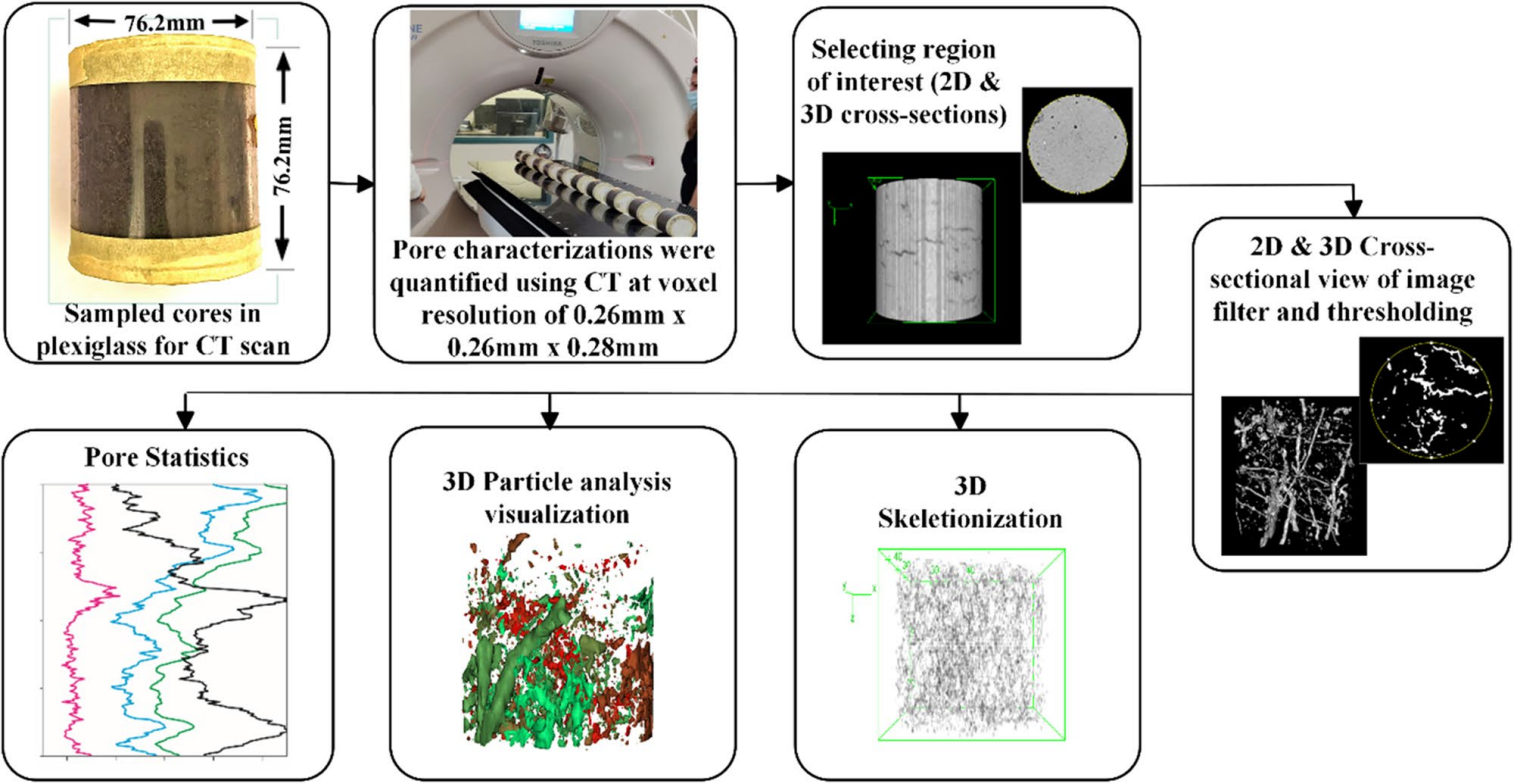
Workflow showing the procedures used in this study for image processing and pore quantification from X-ray micro-computed tomography scanned data.

The mean tortuosity (τ) for the entire soil core was calculated as the ratio of the total actual pore length (*L*_*t*_) to the total Euclidean distance (*L*_*l*_) of all the pores in the soil core:

$$\tau =\frac{\sum_{i=1}^{n}{L}_{t}}{\sum_{i=1}^{n}{L}_{l}}$$

where *i* is the index of a pore branch and *n* is the total number of pore branches in a soil core. In this study, average branch length and τ, were calculated only in the z (vertical) direction. The 3D binary images were used to estimate the FD using the box-counting algorithm in ImageJ. A schematic diagram of the various steps used in XCT analysis is shown in Fig. 6. The image-based soil porosity (cm^3^ cm^−3^) was determined as follows:

$$Porosity ({\text{cm}}^{3} {cm}^{-3}) =\frac{Total\,volume\,of\,pores}{Volume\,of\,ROI}$$

where ROI is the region of interest, that is the cropped CT image used in our analysis.

#### Soil organic carbon and nitrogen assay

Soil samples were sieved through a 0.5 mm sieve for SOC and TN analysis. All visible residues were removed before grinding using a planetary mill pulverisette (Fritsch International). The SOC and TN were determined by dry combustion method^64^ using a Tru-Spec- carbon/hydrogen/nitrogen analyzer (TruSpec; LECO Corporation, St. Joseph, MI, USA).

### Statistical analysis

Analysis of variance (ANOVA) was conducted to compare the effects of different treatments and soil depth on the soil pore characteristics, soil organic carbon, and total nitrogen in the *R* modeling environment RStudio (RStudio, Inc., Boston, MA, USA). Treatment × Depth interactions were also observed using two-way ANOVA. Data were transformed when necessary, using the Box-Cox method. Significance was determined at α = 0.05 level for all statistical analyses in this study. The relationships between XCT-derived soil pore characteristics, SOC, and TN content were analyzed by Pearson's correlation. The statistical significance of Pearson's correlation coefficient was determined at the *p* = 0.05 and *p* = 0.01 levels. Principal component analysis (PCA) of the full dataset was analyzed using the multiple factor analysis (MFA) procedure in RStudio. The PCA was used to subgroup experimental treatments based on the measured XCT-derived soil pore characteristics by generating the eigenvectors of the parameters and component scores for each unit. Each eigenvector loading indicates the direction and magnitude of association between XCT-derived soil pore characteristics and treatments.

## Conclusions

This study observed the effects of long-term (> 10 years) application of different rates of manure and inorganic fertilizer on soil pore characteristics, visualized and quantified using X-ray CT and Image*J* processing. As manure binds the soil particle to form stable aggregates and increases soil moisture absorption and retention, it was observed that the MM treatment improved the TP, Tmacro, and Tmeso at 0–10 and 10–20 cm as compared to MF and HF treatments at Brookings site. While at the Beresford site, the HM treatment increased porosity and macroporosity at 0–10 cm as compared to other treatments, and increased porosity and macroporosity as compared to the HF treatment at 20–30 cm. Because of the direct organic matter addition to the soil, manure treatments also enhanced SOC content at 0–10 and 10–20 cm depths as compared to the inorganic fertilizer treatments at both sites. The SOC was positively correlated to macroporosity and Tmacro and negatively correlated to the BL. The long-term nutrient applications had little impact on the measured parameters in the subsurface (20–40 cm) depths. Overall, the high and medium rates of manure application improved XCT-derived soil pore characteristics, SOC, and TN contents as compared to the inorganic fertilizer and control treatments at 0–10 and 10–20 cm at both sites. This can stabilize the soil structure and improve the water-holding capacity of the soil. However, the long-term application of high manure rates is discouraged as this can adversely impact the environmental. Therefore, we encourage the use of medium manure application in the long term to enhance soil carbon and pore characteristics, enhancing soil health and crop production.

## Data Availability

All data generated or analyzed during this study are included in this published article.

## Abbreviations

XCT: X-ray computed tomography
CK: Control
LM: Low manure
MM: Medium manure
HM: High manure
MF: Medium fertilizer
HF: High fertilizer
BL: Average branch length
FD: Fractal dimension
DA: Degree of anisotropy
