# Author Correction: Cattle manure application for 12 and 17 years enhanced depth distribution of soil organic carbon and X-ray computed tomography-derived pore characteristics

**DOI:** 10.1038/s41598-024-55738-7

**Published:** 2024-02-29

**Authors:** Anuoluwa Ojonoka Sangotayo, Poulamee Chakraborty, Sutie Xu, Sandeep Kumar, Peter Kovacs

**Affiliations:** 1 Department of Agronomy, Horticulture, and Plant Science, South Dakota State University, Brookings, SD 57007 USA; 2 Department of Plant, Soil, and Microbial Sciences, Michigan State University, East Lansing, MI 48824 USA

Correction to: *Scientific Reports* 10.1038/s41598-023-50110-7, published online 27 December 2023

In the original version of this Article Sandeep Kumar was incorrectly affiliated with ‘National Institute of Food and Agriculture, United States Department of Agriculture, Bucyrus, KS 66013, USA’. The correct affiliation is listed below.

Department of Agronomy, Horticulture, and Plant Science, South Dakota State University, Brookings, SD 57007, USA

Also, Sandeep Kumar was omitted as a corresponding author. Correspondence and requests for materials should also be addressed to kaundal@gmail.com.

The original Article has been corrected.

